# Transfer of breast milk IgA to infants after oral bivalent norovirus vaccination of post-partum women

**DOI:** 10.1038/s41541-025-01361-0

**Published:** 2026-01-14

**Authors:** Molly R. Braun, Lam-Quynh Nguyen, Becca A. Flitter, Nicholas J. Bennett, Darreann Carmela M. Hailey, Colin A. Lester, Elena D. Neuhaus, Kirsten Marx, Nick P. D’Amato, Kayan Tam, Marcela F. Pasetti, Sean N. Tucker, James F. Cummings

**Affiliations:** 1 Vaxart, Inc., South San Francisco, CA USA; 2 Center for Vaccine Development, University of Maryland School of Medicine, Baltimore, MD USA

**Keywords:** Diseases, Health care, Immunology, Medical research, Microbiology

## Abstract

Norovirus can cause severe and potentially fatal gastroenteritis in infants. Mucosal vaccination of breastfeeding women may promote infant protection by enriching antibody responses in consumed breast milk. Here, we report a double-blind, placebo-controlled phase 1 trial in South Africa (SANCTR: DOH-27-072023-7893) to evaluate a single-dose oral bivalent vaccine against norovirus genotypes GI.1 and GII.4 in post-partum breastfeeding women. Safety and reactogenicity (primary outcome), breast milk and serum norovirus-specific antibodies (primary outcome), and passive transfer of antibodies to infants as measured in infant stool (exploratory outcome) were assessed. The vaccine was safe and well tolerated with similar reports of mild or moderate adverse events between placebo (*n* = 16) and vaccine groups (5 × 10^10^ or 1 × 10^11^ IU/genotype, *n* = 30/group). Functional norovirus-specific breast milk and serum antibodies were significantly enriched in vaccinated groups. Norovirus-specific IgA in infant stool increased post-vaccination and positively correlated with breast milk IgA, indicating passive transfer. Thus, oral vaccination of breastfeeding women generates robust mucosal and systemic functional maternal antibodies. Our study presents a promising vaccination strategy to provide mucosal anti-norovirus immunity to infants.

## Introduction

Norovirus is a highly contagious virus belonging to the family *Caliciviridae* and is a major cause of acute gastroenteritis (AGE) worldwide^1^. Infection is typically characterized by the sudden and severe onset of nausea, vomiting, abdominal pain, and diarrhea^2^, which can lead to significant morbidity and mortality in vulnerable populations; notably in children from under-resourced areas^3^. Norovirus is estimated to cause more than 200,000 deaths annually worldwide^4^. A 2013 report estimated that 70,000 deaths occurring from infection with caliciviruses are in children <5 years of age^5^. Global surveillance of norovirus in children between 2016-2020 revealed that more than 50% of infections could be attributed to GII.4 Sydney^6^.

There are no approved vaccines or antivirals for norovirus. Treatment is limited to supportive care aiming to alleviate dehydration and electrolyte imbalances associated with AGE^7^. A previous study of norovirus infection revealed that mucosal immune responses correlated with prevention of disease and viral shedding^8^. Therefore, it is likely that successful vaccination strategies must generate robust mucosal immunity. A previous attempt to develop a childhood norovirus vaccine composed of virus-like particles (VLPs) administered intramuscularly by injection demonstrated safety and serum-based immunogenicity, although mucosal immune responses were not shown^9,10^. Recently, a phase 2b clinical trial using this injected vaccine in infants failed to demonstrate protective efficacy against moderate or severe acute AGE and showed no clinical benefit across primary or secondary endpoints^11^. It is unknown why vaccine efficacy was low, though it is possible that this parenterally delivered vaccine approach did not elicit sufficient mucosal immunity associated protection. In contrast, in a phase 2 norovirus GI.1 challenge study, we showed the induction of vaccine-induced mucosal immunity and a correlation between fecal IgA and protection from norovirus infection after vaccination with an orally-administered non-replicating adenovirus-vectored vaccine, VXA-G1.1-NN^12^.

While direct vaccination of infants has been utilized for previous norovirus trials^10^, passive transfer of polyclonal mucosal antibodies to infants via breast milk may be more effective and practical for preventing AGE in infants^13^. Adults likely possess norovirus-specific immune memory from prior environmental exposure. Thus, oral vaccination may enhance and mature the existing norovirus-specific antibody repertoire, resulting in a transfer of affinity-matured breast milk antibodies that couldn’t be achieved by vaccination of a naïve population, like infants. While infants possess some antigen-specific IgG acquired in utero, protection against infectious diseases is extended through consumption of breast milk containing ample secretory IgA (S-IgA), some IgG, and other immune components^14^. It was observed that infants consuming breast milk with norovirus-specific IgA had reduced diarrheal symptoms during infection compared to those consuming breast milk without these antibodies^13^. Enhancing or extending infant protection by increasing breast milk norovirus-specific antibodies could have a significant impact on norovirus-related diarrheal disease and mortality in infants, while also reducing community transmission within close contact settings.

GI and GII are the two most common norovirus genogroups that cause human disease. GI.1 and GII.4 have been associated with large outbreaks, with GII.4 accounting for the majority of global AGE outbreaks in the last 20 years^15^. A model of population-level impacts of norovirus vaccines stress that inclusion of the GII.4 genotype is critical for reducing the incidence of norovirus, particularly in pediatric and older-aged individuals^16^. Our group developed a thermostable, orally-delivered, bivalent norovirus vaccine encoding the viral capsid protein, VP1, of GI.1 (VXA-G1.1-NN) and GII.4 (VXA-G2.4-NS)^17^. This vaccine is an E1/E3-deleted replication-incompetent recombinant human adenovirus type 5 (rAd5) vector that expresses the vaccine-antigen gene of interest alongside with a double-stranded ribonucleic acid hairpin molecular adjuvant, activating immune responses in same cell as antigen expression. The rAd5 is formulated into enterically coated thermostable tablets and is delivered to the intestinal ileum via swallowing. This vaccine platform has demonstrated safety, tolerability, and immunogenicity (in serum, saliva, nasal lining fluid [NLF], and feces) in United States-based adults aged 18–80^18–22^ and this specific bivalent vaccine combination previously demonstrated safety and immunogenicity against norovirus GI.1 and GII.4 in phase 1 and 2 clinical trials (NCT03897309, NCT05626803, NCT06944717) in adults aged 18-80. Further, we demonstrated the protective efficacy of oral VXA-G1.1-NN against infection in a human GI.1 phase 2b challenge model, where we found that fecal norovirus-specific binding IgA and serum blocking antibodies were correlated with protection against infection^12^. In this study, significant reductions of viral shedding in feces compared to placebo were observed, indicating that vaccination might lessen transmission as well as symptomatic disease^12^. Although we have shown induction of immune responses at distal mucosal sites, it was unknown if vaccine-specific antibodies could be generated in the breast milk of women from under-resourced countries, and if those antibody responses could be identified in the stool of paired infants. As a proof-of-concept study, we conducted a phase 1, double-blind, placebo-controlled trial to evaluate the safety, tolerability, and immunogenicity of a single-dose bivalent GI.1/GII.4 vaccine administered orally to healthy breastfeeding women. The vaccine was found to be safe and well-tolerated. Notably, vaccine-induced IgA responses were detected in the breast milk of vaccinated participants and the stool of paired infants, demonstrating the potential of this oral GI.1/GII.4 bivalent vaccine approach to provide passive norovirus immunity to breastfeeding infants.

## Results

### Study design and safety of an oral bivalent norovirus vaccine

**Fig. 1:**
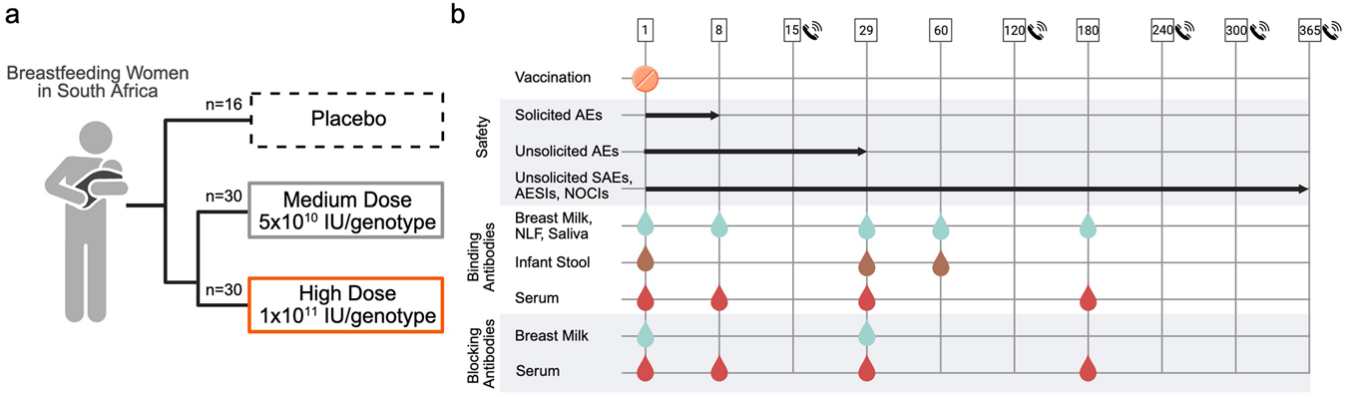
Clinical trial design and sample collection. **a** Description of participant vaccination groups. **b** Overview of trial timelines, biosample collections, and immune analyses performed. Numbers represent study days. Study visits conducted only by phone are noted with a phone symbol. IU *Infectious units*, AE *adverse event*, SAE *serious adverse event*, AESI *adverse event of special interest*, NOCI *new onset of chronic illness*, NLF *nasal lining fluid*. Figure created in https://BioRender.com.

In this phase 1 study, we evaluated the safety and immunogenicity of an oral bivalent norovirus vaccine in breastfeeding women. From October 2^nd^, 2023, to December 7^th^, 2023, 100 women were screened across five clinical sites in South Africa, and 76 participants were dosed after being randomly assigned to treatment groups: 16 in placebo, 30 in the medium-dose group (5 × 10^10^ IU/genotype), and 30 in the high-dose group (1 × 10^11^ IU/genotype) (Fig. 1a, Supplementary Fig. 1). The active period, defined as day 1 to day 29, concluded for all participants between November 22^nd^, 2023, and January 11^th^, 2024. The follow-up period, defined as day 30 to day 365, concluded for all participants between October 25^th^, 2024, and December 13^th^, 2024. The participants had an overall mean age of 27.2, a mean BMI of 28.4, and a mean time post-partum of 192 days, with no statistical differences between dose groups (Table 1). Almost all participants reported their ethnicity as Black, with five participants reporting their ethnicity as ‘Other – Colored’ or ‘Other – South African Colored.’ All dosed participants were included in the safety and intention-to-treat analysis. All participants who had at least one pre-vaccination immunogenicity result and one valid immunogenicity result after day 1 were included in the Immunogenicity Analysis Set. Primary safety outcomes included solicited symptoms through day 8 post vaccination and unsolicited symptoms through day 29, while serious adverse events (SAEs), adverse events of special interest (AESIs), and new onsets of chronic illness (NOCIs) were monitored through 12 months post vaccination (secondary outcome). Immunogenicity was assessed by the generation of norovirus-specific IgA in breast milk and serum through either day 29 (primary outcome) and day 180 (secondary outcome). Secondary outcomes included functional serum antibody titers, norovirus-specific breast milk and serum IgA through day 180, serum IgG, saliva IgA, and NLF IgA. Exploratory outcomes included detection of norovirus-specific infant stool IgA and functional breast milk IgA. Functional antibodies were measured by blocking ability in a surrogate neutralizing assay (Fig. 1b**)**.

**Table 1:** Demographic characteristics of patients by cohort

Parameter	Statistic	Placebo cohort	Medium dose cohort	High dose cohort	Medium + high dose cohort	Overall
		(*N* = 16)	(*N* = 30)	(*N* = 30)	(*N* = 60)	(*N* = 76)
Age, years (SD)		25.4 (5.2%)	28.6 (5.7%)	26.8 (6.5%)	27.7 (6.1%)	27.2 (6.0%)
Sex, *n* (%)	Female	16 (100%)	30 (100%)	30 (100%)	60 (100%)	76 (100%)
Childbearing Potential, *n* (%)	Yes	16 (100%)	29 (96.6%)	30 (100%)	60 (100%)	76 (100%)
	No	0	1 (3.3)^1^	0	0	0
Race, *n* (%)	Black	15 (93.8%)	29 (96.7%)	27 (90.0%)	56 (93.3%)	71 (93.4%)
	White	0	0	0	0	0
	Other^2^	1 (6.3%)	1 (3.3%)	3 (10.0%)	4 (6.7%)	5 (6.6%)
BMI, kg/m^2^ (SD)		25.8 (5.4%)	29.5 (7.4%)	28.8 (7.5%)	29.2 (7.4%)	28.4 (7.1%)
Metabolism and nutrition disorders, *n* (%)	Iron deficiency	1 (6.3%)	0	0	0	1 (1.3%)
	Obesity	3 (18.8%)	5 (16.7%)	2 (6.7%)	7 (11.7%)	10 (13.2%)
	Overweight	0	1 (3.3%)	1 (3.3%)	2 (3.3%)	2 (2.6%)
Pregnancy conditions, *n* (%)	Pre-eclampsia	0	1 (3.3%)	0	1 (1.7%)	1 (1.3%)
Reproductive system and breast disorders, *n* (%)	Abnormal uterine bleeding	1 (6.3%)	0	0	0	1 (1.3%)
	Amenorrhea	0	1 (3.3%)	0	1 (1.7%)	1 (1.3%)
Surgical and medical procedures, *n* (%)	Cesarean section	0	4 (13.3%)	1 (3.3%)	5 (8.3%)	5 (6.6%)
	Female sterilization	0	1 (3.3%)	0	1 (1.7%)	1 (1.3%)
	Umbilical hernia repair	0	0	1 (3.3%)	1 (1.7%)	1 (1.3%)
Time post-partum/Infant Age	Days	191.9 (73.2%)	179.4 (83.1%)	205.8 (80.1%)	192.6 (82.3%)	192.4 (80.0%)
Breast milk provided through six months *n* (%)		16 (100.0%)	27 (90.0%)	27 (90.0%)	54 (90.0%)	70 (92.0)

^1^One subject underwent tubal ligation during c-section.

^2^Self-reported as ‘Other’, including four reporting ‘Other – Colored’ and one reporting as ‘Other – South African Colored’. *BMI* body mass index, *SD* standard deviation.

All solicited symptoms were of mild to moderate severity, and the number and severity of symptoms were not dose-dependent. No adverse events beyond grade 2 were reported. A total of six participants (37.5%) in the placebo group, 16 participants (53.3%) in the medium-dose group, and 13 participants (43.3%) in the high-dose group reported symptoms. Overall, the most frequently reported solicited symptoms were headache (placebo, *n* = 4, 25%; medium, *n* = 6, 20%; high, *n* = 8, 26.7%) and nausea (placebo, *n* = 1, 6.3%; medium, *n* = 5, 16.7%; high, *n* = 6, 20%). No solicited symptoms of reactogenicity were reported beyond day 8 (Table 2). A similar percentage of participants in the placebo and vaccinated groups reported TEAE through day 29, including 2 participants (12.5%) in the placebo group, and 3 participants (10%) in each of the medium and high-dose groups (Supplementary Table 1). No SAEs, AESIs, or NOCIs were reported through day 365.

**Table 2:** Solicited symptoms from day 1 to 8

Solicited Symptom/Grade^1^	Placebo cohort	Medium dose cohort	High dose cohort	Medium + high dose cohorts	Overall
	(*N* = 16)	(*N* = 30)	(*N* = 30)	(*N* = 60)	(*N* = 76)
**Subjects with Any Solicited Symptom, n (%)**	**6 (37.5%)**	**16 (53.3%)**	**13 (43.3%)**	**29 (48.3%)**	**35 (46.1%)**
Mild	5 (31.3%)	15 (50.0%)	8 (26.7%)	23 (38.3%)	28 (36.8%)
Moderate	1 (6.3%)	1 (3.3%)	5 (16.7%)	6 (10.0%)	7 (9.2%)
Severe	0	0	0	0	0
Life-threatening	0	0	0	0	0

Fever^2^, *n* (%)	0	2 (6.7%)	2 (6.7%)	4 (6.7%)	4 (5.3%)
Mild	0	2 (6.7%)	2 (6.7%)	4 (6.7%)	4 (5.3%)
Moderate	0	0	0	0	0
Severe	0	0	0	0	0
Life-threatening	0	0	0	0	0

Headache, *n* (%)	4 (25.0%)	6 (20.0%)	8 (26.7%)	14 (23.3%)	18 (23.7%)
Mild	3 (18.8%)	6 (20.0%)	4 (13.3%)	10 (16.7%)	13 (17.1%)
Moderate	1 (6.3%)	0	4 (13.3%)	4 (6.7%)	5 (6.6%)
Severe	0	0	0	0	0
Life-threatening	0	0	0	0	0

Myalgia (Muscle Pain), *n* (%)	0	3 (10.0%)	2 (6.7%)	5 (8.3%)	5 (6.6%)
Mild	0	2 (6.7%)	1 (3.3%)	3 (5.0%)	3 (3.9%)
Moderate	0	1 (3.3%)	1 (3.3%)	2 (3.3%)	2 (2.6%)
Severe	0	0	0	0	0
Life-threatening	0	0	0	0	0

Abdominal Pain, *n* (%)	2 (12.5%)	1 (3.3%)	2 (6.7%)	3 (5.0%)	5 (6.6%)
Mild	2 (12.5%)	1 (3.3%)	1 (3.3%)	2 (3.3%)	4 (5.3%)
Moderate	0	0	1 (3.3%)	1 (1.7%)	1 (1.3%)
Severe	0	0	0	0	0
Life-threatening	0	0	0	0	0

Anorexia, *n* (%)	1 (6.3%)	0	2 (6.7%)	2 (3.3%)	3 (3.9%)
Mild	1 (6.3%)	0	2 (6.7%)	2 (3.3%)	3 (3.9%)
Moderate	0	0	0	0	0
Severe	0	0	0	0	0
Life-threatening	0	0	0	0	0

Nausea, *n* (%)	1 (6.3%)	5 (16.7%)	6 (20.0%)	11 (18.3%)	12 (15.8%)
Mild	1 (6.3%)	5 (16.7%)	5 (16.7%)	10 (16.7%)	11 (14.5%)
Moderate	0	0	1 (3.3%)	1 (1.7%)	1 (1.3%)
Severe	0	0	0	0	0
Life-threatening	0	0	0	0	0

Vomiting, *n* (%)	0	1 (3.3%)	0	1 (1.7%)	1 (1.3%)
Mild	0	1 (3.3%)	0	1 (1.7%)	1 (1.3%)
Moderate	0	0	0	0	0
Severe	0	0	0	0	0
Life-threatening	0	0	0	0	0

Diarrhea, *n* (%)	1 (6.3%)	4 (13.3%)	4 (13.3%)	8 (13.3%)	9 (11.8%)
Mild	1 (6.3%)	4 (13.3%)	3 (10.0%)	7 (11.7%)	8 (10.5%)
Moderate	0	0	1 (3.3%)	1 (1.7%)	1 (1.3%)
Severe	0	0	0	0	0
Life-threatening	0	0	0	0	0

Malaise/Fatigue, *n* (%)	2 (12.5%)	2 (6.7%)	3 (10.0%)	5 (8.3%)	7 (9.2%)
Mild	2 (12.5%)	2 (6.7%)	2 (6.7%)	4 (6.7%)	6 (7.9%)
Moderate	0	0	1 (3.3%)	1 (1.7%)	1 (1.3%)
Severe	0	0	0	0	0
Life-threatening	0	0	0	0	0

^1^The following guidance was provided to participants: Mild; awareness of a symptom but the symptom is easily tolerated. Moderate; discomfort enough to cause interference with usual activity. Severe; incapacitating – unable to perform usual activities – requires absenteeism or bed rest. Life Threatening; ER/AE visit or hospitalization.

^2^Fever is defined as body temperature measured: mild (100.4 °F to 101.1 °F), moderate (101.2 °F to 102.0 °F), severe (102.1 °F to 104.0 °F) and potentially life-threatening (>104.0 °F). Abbreviations: N/A.

### Oral vaccination generated norovirus-specific antibodies in breast milk that were passively transferred to infants

**Fig. 2:**
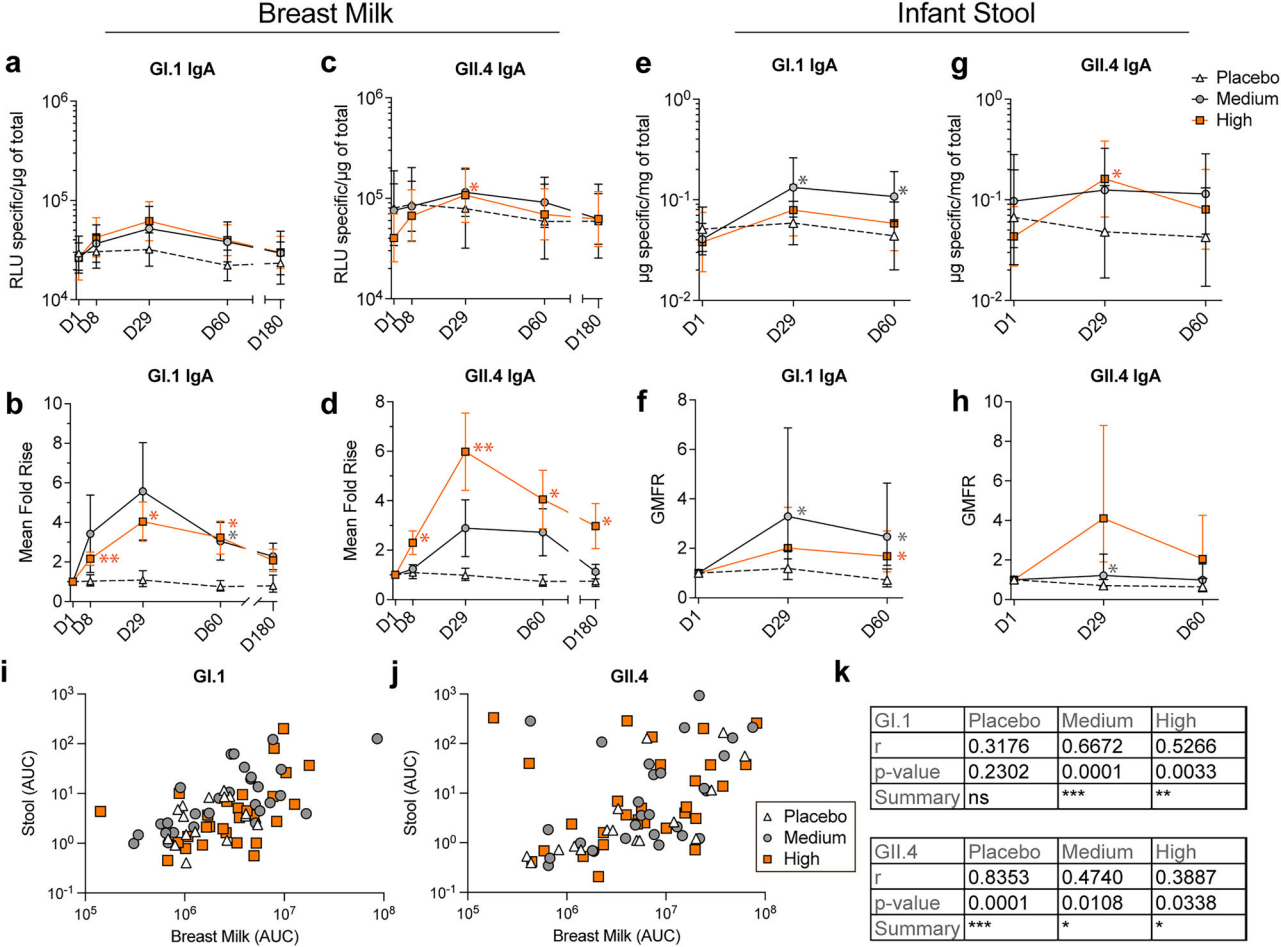
Oral vaccination induced durable breast milk antibody responses in participants that was transferred to infants. **a** Geometric mean +/- 95% CI of breast milk anti-GI.1 IgA normalized to total IgA per sample at indicated times post vaccination and **b** mean fold rise +/- SEM of over day 1. **c** Geometric mean +/- 95% CI of breast milk anti-GII.4 IgA normalized to total IgA per sample at indicated times post vaccination and **d** mean fold rise +/- SEM over day 1. **e** Geometric mean + /- 95% CI of infant stool anti-GI.1 IgA normalized to total IgA per sample at indicated times post vaccination and **f** geometric mean fold rise +/- SEM of over day 1. **g** Geometric mean + /- 95% CI of infant stool anti-GII.4 IgA normalized to total IgA per sample at indicated times post vaccination and **h** geometric mean fold rise +/- 95% CI over day 1. Paired **i** GI.1 and **j** GII.4 breast milk AUC to infant stool AUC for each participant. **k** Summary of non-parametric Spearman, two-tailed, correlation analysis. Statistical tests **a**–**h**: Mixed-effect analysis with multiple comparisons, and Uncorrected Fisher’s Least Significant Difference (LSD) comparing each timepoint to placebo. AUC *area under the curve*, RLU *relative light units*.

Breast milk IgA responses to GI.1 and GII.4 VLPs were quantified using Meso Scale Discovery (MSD) technology on days 1, 8, 29, 60, and 180 post-vaccination and normalized to total IgA concentration. There were no significant differences in baseline breast milk GI.1-specific IgA concentrations across groups (day 1 geometric mean [RLU of specific IgA/µg of total IgA]; placebo, 29393.5; medium, 26300.4; high, 26309.0, *p* > 0.21) (Fig. 2a). Vaccinated medium and high-dose groups’ highest levels of GI.1-specific IgA were observed on day 29 post vaccination, with a mean fold rise of 5.57 (ns) and 4.04 (*p* = 0.018), respectively, compared to 1.40 in the placebo group (Fig. 2b, Supplementary Table 2). Breast milk GI.1-specific IgA remained above baseline through day 180 with a mean fold rise of 2.28 and 2.08 in the medium and high-dose groups compared to 1.35 in the placebo group (Fig. 2b, Supplementary Table 2). Similarly, there were no significant differences in baseline breast milk GII.4-specific IgA concentrations across groups (day 1 geometric mean RLU of specific IgA/µg of total IgA; placebo, 79636.1; medium, 75811.2; high, 40270.6, *p* > 0.068) (Fig. 2c). The highest levels of breast milk GII.4-specific IgA were observed at day 29 with mean fold rises of 2.89 (ns) and 5.99 (p = 0.004) in the medium and high-dose groups, respectively, compared to 1.08 in the placebo group (Fig. 2d, Supplementary Table 2). Breast milk GII.4-specific IgA remained above baseline through day 180 in the high-dose group with a mean fold rise of 2.98, compared to placebo and medium-dose groups with fold rises of 0.87 and 1.13, respectively (Fig. 2d). Vaccine-induced breast milk antibody levels were not dependent on BMI, age, or time post-partum (Supplementary Fig. 2a–c). These results demonstrate vaccine-induced breast milk IgA in vaccinated, but not placebo groups, with antibody levels remaining above baseline six months after vaccination in the high dose group.

To discern whether vaccine-induced breast milk IgA was passively transferred to the breastfed infants, norovirus-specific stool IgA was measured from the infants of the vaccinated participants on days 0, 29, and 60. GI.1- and GII.4-specific IgA were measured and normalized to total IgA concentration in infant stool. There were no significant differences in baseline infant stool GI.1-specific IgA concentrations across groups (day 1 geometric mean [mg of specific/g of total IgA]; placebo, 0.053; medium, 0.041; high, 0.033, *p* > 0.35) (Fig. 2e, Supplementary Table 2). The highest levels of infant stool GI.1-specific IgA were observed at day 29 with geometric mean fold rises of 3.29 (*p* = 0.033) and 2.01 (ns) in the medium and high-dose groups, compared to 1.19 in the placebo group (Fig. 2f, Supplementary Table 2). Likewise, there were no significant differences in baseline infant stool GII.4-specific IgA concentrations across groups (day 1 geometric mean [mg of specific/g of total IgA]; placebo 0.052; medium, 0.097; high, 0.039, *p* > 0.067) (Fig. 2g, Supplementary Table 2). The highest levels of infant stool GII.4-specific IgA were observed at day 29 for medium and high-dose groups with geometric mean fold rises of 1.22 (*p* = 0.05), and 4.10 (ns), respectively, compared to placebo at 0.71 (Fig. 2h, Supplementary Table 2). Three infants had stool samples with >100-fold rises; one medium dose sample at day 29 for GI.1 and two high dose samples at days 29 and 60 for GII.4, leading to large error bars. None of these infants reported norovirus-like disease symptoms. Infant stool antibody levels were not dependent on infant age (Supplementary Fig. 2d). Overall, a consistent trend of increased GI.1 and GII.4-specific IgA was observed in the stool from paired infants of vaccinated participants at days 29 and 60.

To relate the norovirus-specific IgA content in infant stool samples with norovirus-specific IgA in breast milk, we compared the area under the curve (AUC) of breast milk IgA (days 1-8-29-60) and infant stool IgA (days 1-29-60) from each dyad using Spearman correlations (Fig. 2i, j, Supplementary Table 2). A significant positive association was found between the quantities of maternal milk and infant stool GI.1-specific IgA in the medium and high-dose groups, but not for the placebo group (p-value; placebo, 0.23; medium, 0.0001; high, 0.0033) and for GII.4-specific IgA in all dose groups, including the placebo group (p-value; placebo, 0.0001; medium, 0.0108; high 0.0338) (Fig. 2k, Supplementary Table 2). To further support the hypothesis that norovirus specific antibodies found in infant stool originated from breast milk, we examined the proportion of norovirus-specific IgA1 and IgA2 post vaccination in the breast milk and infant stool, using the high-dose group as a subset, and found that GI.1 and GII.4-specific IgA1:IgA2 ratios increased on day 29 in both breast milk and infant stool (Supplementary Fig. 3a, b). These results suggest that observed increases in norovirus-specific infant stool IgA originated from breast milk, as the temporal distribution of IgA subclasses was mirrored in the two biosamples.

### Oral vaccination generated functional norovirus-specific antibodies in breast milk

**Fig. 3:**
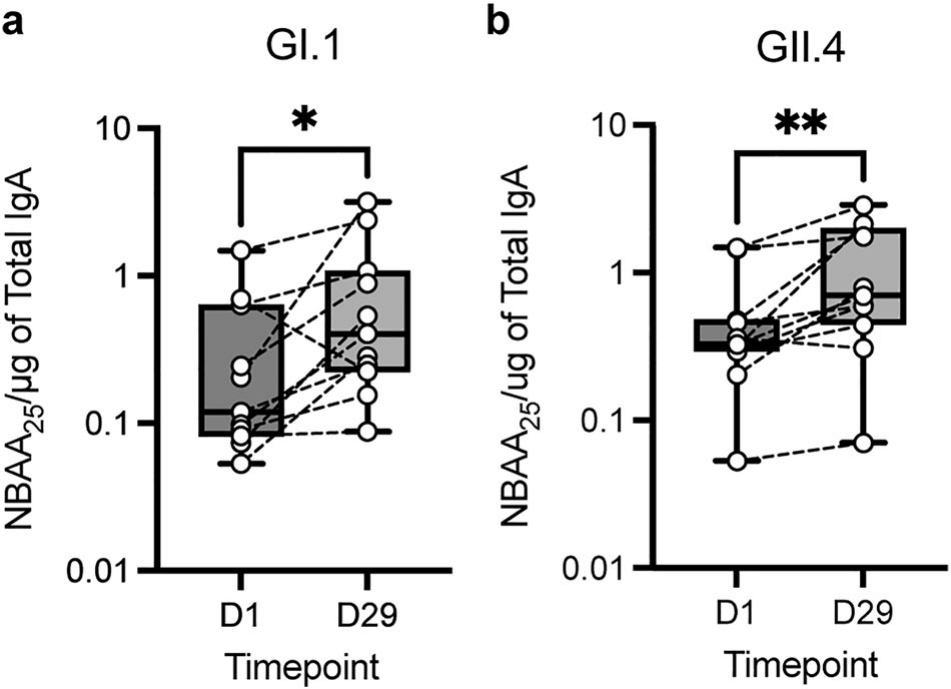
Oral vaccination induced functional breast milk antibodies. Purified breast milk antibodies from samples with ≥ twofold rises in binding antibody concentration were tested for blocking abilities specific to **a** GI.1 and **b** GII.4 VLPs and normalized to total IgA. Wilcoxon paired t-test, *n* = 11. NBAA *norovirus blocking antibody assay*.

In addition to identifying norovirus-specific breast milk IgA, we sought to determine whether oral vaccination induced functional breast milk antibody responses. In a previous human challenge study, we found that functional serum antibodies, as measured by the norovirus blocking antibody assay (NBAA), a surrogate neutralization assay, were correlated with protection from norovirus infection^12^. Therefore, the presence of functional antibodies in breast milk may play a crucial role in providing protection to breastfed infants. IgA was purified from 11 paired pre- and post-vaccination breast milk samples that had a greater than twofold increase in both GI.1 and GII.4-specific IgA across the medium and high-dose groups. Purified IgA preparations were incubated with GI.1 and GII.4 VLPs then added to plates precoated with the HBGAs Le^b^ or H Type 3 (carbohydrate receptors of GI.1 and GII.4, respectively). The 25% blocking titers (NBAA_25_) of the purified IgA were determined for days 1 and 29 and normalized to total IgA. The traditional readout for this assay using a serum matrix is the 50% blocking titer; however, due to limited recovery of breast milk antibodies, NBAA_25_ was used as the threshold for functional activity. In most cases, individual subjects’ isolated breastmilk antibody blocking activity increased from days 1 to 29 for both GI.1 and GII.4 with mean fold rises of 2.51 (*p* = 0.024) and 2.14 (*p* = 0.0029) (Fig. 3a, b). This observation indicates that this vaccine approach has the capacity to induce functional antibodies in breast milk that block norovirus:carbohydrate interactions.

### Oral vaccination generated norovirus-specific binding and functional antibodies in serum

**Fig. 4:**
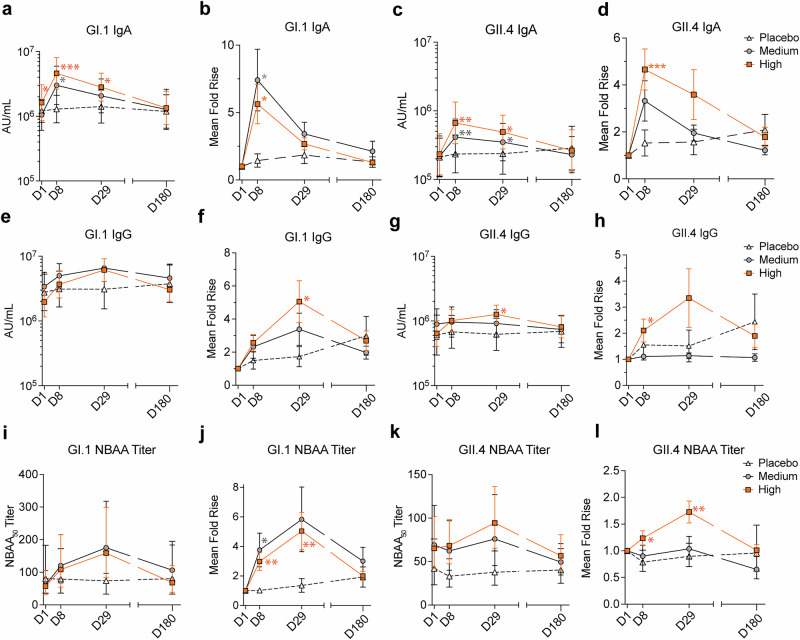
Oral vaccination induced serum antibody responses. **a** Geometric mean +/- 95% CI of serum anti-GI.1 IgA at indicated times post vaccination and **b** mean fold rise of over day 1. **c** Geometric mean + /- 95% CI of serum anti-GII.4 IgA at indicated times post vaccination and (**d**) mean fold rise of over baseline. **e** Geometric mean +/- 95% CI of serum anti-GI.1 IgG at indicated times post vaccination and (**f**) mean fold rise of over day 1. **g** Geometric mean +/- 95% CI of serum anti-GII.4 IgG at indicated times post vaccination and **h** mean fold rise of over day 1. **i** Geometric mean + /- 95% CI of serum GI.1 NBAA_50_ titer at indicated times post vaccination and **j** mean fold rise of over day 1. **k** Geometric mean + /- 95% CI of serum GII.4 NBAA_50_ at indicated times post vaccination and **l** mean fold rise over day 1. Statistical tests a-l: Mixed-effect analysis with multiple comparisons, and Uncorrected Fisher’s Least Significant Difference (LSD) comparing each timepoint to placebo. AU *arbitrary units*, AUC *area under the curve,* NBAA *norovirus blocking* *antibody assay*.

Serum GI.1 and GII.4-specific IgA and IgG responses on days 1, 8, 29, and 180 were quantified using MSD assays. There was a significant difference between the baseline serum GI.1-specific IgA concentrations in placebo and high-dose groups (*p* = 0.042) while no differences were found between the medium-dose group and any other group (day 1, geometric mean (AU/mL); placebo, 1209786.5; medium, 1050560.6; high, 1625693.1, *p* > 0.20 for all other comparisons). The highest levels of GI.1-specific serum IgA were observed at day 8 for medium and high-dose groups, achieving mean fold rises of 7.41 (*p* = 0.016) and 5.63 (*p* = 0.011), respectively, compared to placebo at 1.44 (Fig. 4a, b, Supplementary Table 2). There were no significant differences in baseline serum GII.4-specific IgA concentrations across groups (day 1 geometric mean AU/mL; placebo, 211741.7; medium, 217289.1; high, 233243.3, *p* > 0.089). The highest levels of GII.4-specific serum IgA were observed at day 8 with mean fold rises of 3.32 (ns) and 4.66 (*p* = 0.0042) in the medium and high-dose groups, compared to 1.53 in the placebo group (Fig. 4c, d, Supplementary Table 2). Both GI.1- and GII.4-specific IgA levels decreased thereafter, returning to baseline at day 180.

There were no significant differences in baseline serum GI.1-specific IgG levels among groups (day 1 geometric mean [AU/mL]; placebo, 2765544.4; medium, 3397392.0; high, 1964195.4, *p* > 0.36). The highest levels of GI.1-specific IgG were observed at day 29 for both the medium and high-dose groups, reaching mean fold rises of 3.38 (ns) and 5.06 (*p* = 0.023), respectively, compared to 1.73 for the placebo group (Fig. 4e, f, Supplementary Table 2). There were no significant differences in baseline serum GII.4-specific IgG concentrations (day 1 geometric mean [AU/mL]; placebo 605754.7; medium, 893913.0; high, 641462.6, *p* > 0.32). The highest levels of GII.4 IgG were observed at day 29 with a mean fold rise of 3.35 (*ns*) for the high-dose group, compared to 1.14 and 1.51 for the medium and placebo groups, respectively (Fig. 4g, h, Supplementary Table 2).

There were no significant differences in serum baseline NBAA GI.1 titers (day 1 geometric mean titers; placebo, 80.5; medium-dose, 61.6, high-dose, 57.4, *p* > 0.56). The highest levels of NBAA GI.1 responses were observed at day 29 in the medium and high-dose groups with mean fold rises of 5.83 (ns, *p* = 0.054) and 5.04 (*p* = 0.0098), respectively, compared to placebo at 1.36 (Fig. 4i, j**)**. There were no significant differences in baseline NBAA GII.4 titers (day 1 geometric mean titers; placebo, 65.22; medium-dose, 42.05, high-dose, 69.10, *p* > 0.46). The highest levels of NBAA GII.4 responses were observed at day 29 in the high-dose group with a mean fold rise of 1.73 (*p* = 0.0023). The medium-dose group mean fold rise was modest at 1.2 (ns) compared to placebo at 0.98. (Fig. 4k, l, Supplementary Table 2**)**. These results demonstrate the production of functional systemic antibodies following vaccination with this oral vaccine.

### Oral vaccination generated norovirus-specific salivary and nasal antibodies

**Fig. 5:**
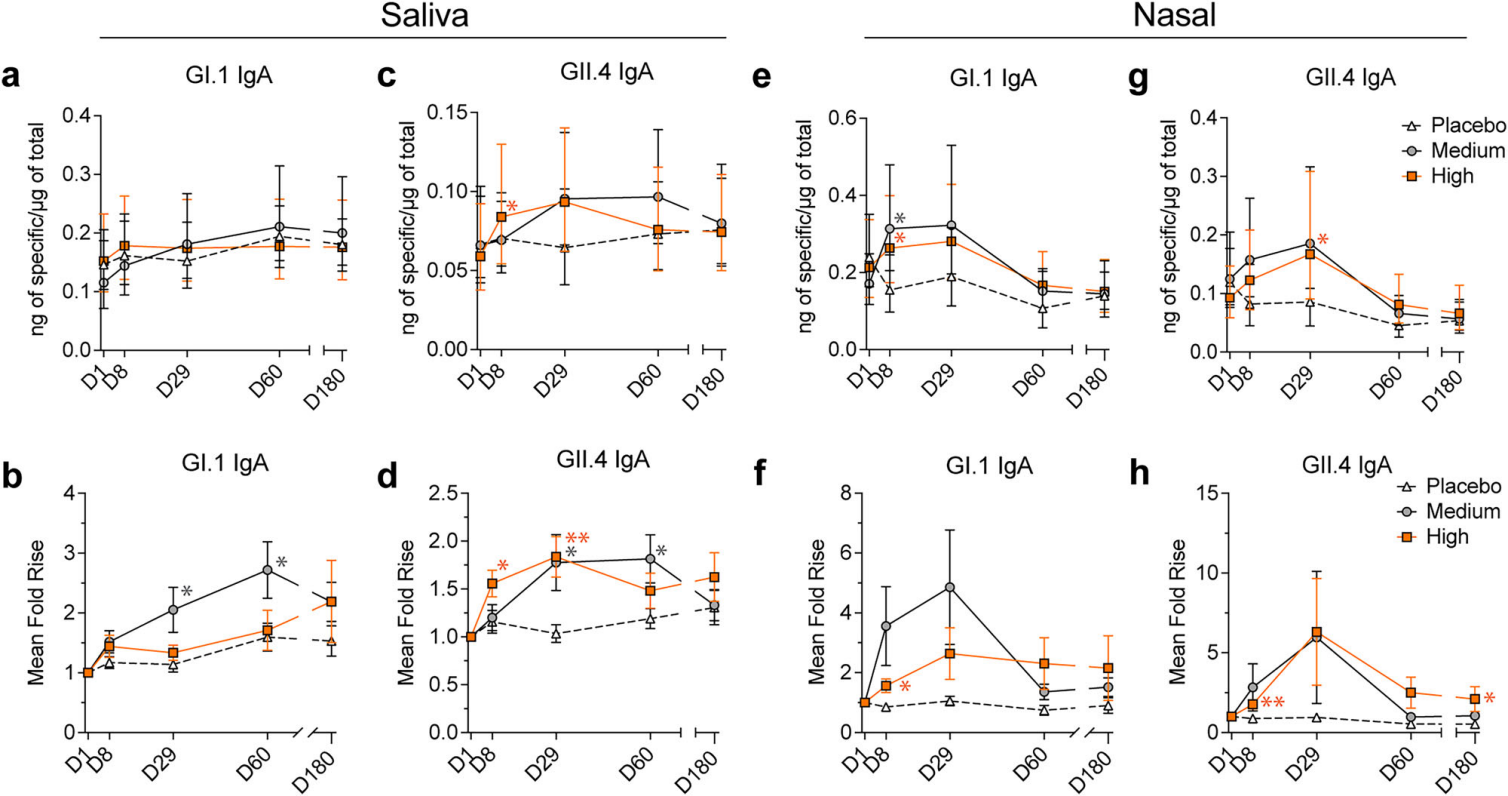
Mucosal IgA is induced in saliva and NLF after oral vaccination to the small intestine. **a** Geometric mean +/- 95% CI of salivary anti-GI.1 IgA at indicated times post vaccination and **b** mean fold rise +/- SEM of over day 1. **c** Geometric mean +/- 95% CI of salivary anti-GII.4 IgA at indicated times post vaccination and **d** mean fold rise +/- SEM of over day 1. **e** Geometric mean +/- 95% CI of nasal anti-GI.1 IgA at indicated times post vaccination and **f** mean fold rise +/- SEM of over day 1. **g** Geometric mean +/- 95% CI of nasal anti-GII.4 IgA at indicated times post vaccination and **h** mean fold rise +/- SEM of over day 1. Statistical tests **a**–**h**: Mixed-effect analysis with multiple comparisons, and Uncorrected Fisher’s Least Significant Difference (LSD) comparing each timepoint to placebo. NLF *nasal lining fluid*.

GI.1 and GII.4-specific salivary and nasal IgA to GI.1 and GII.4 on days 1, 8, 29, 60 and 180 were quantified using MSD assays. There were no significant differences in baseline salivary GI.1-specific IgA concentrations across groups (day 1 geometric mean [ng of specific/µg of total IgA]; placebo, 0.146; medium, 0.115; high, 0.152, *p* ≥ 0.27). GI.1-specific IgA in the medium-dose group increased over time with mean fold rises of 2.05 (*p* = 0.028), 2.72 (*p* = 0.039), and 2.19 (ns) on days 29, 60, and 180, respectively (Fig. 5a, b, Supplementary Table 2), while the high-dose group had minimal levels of detectable antibodies. The same trend was observed in the NLF. There were no significant differences in baseline nasal GI.1-specific IgA concentrations across groups (day 1 geometric mean [ng of specific/µg of total IgA]; placebo, 0.239; medium, 0.171; high, 0.213, *p* ≥ 0.30). GI.1-specific nasal IgA increased over time in the medium-dose group with mean fold rises of 3.56 (*p* = 0.050) and 4.86 (ns, *p* = 0.057) for days 8 and 29, respectively, while the high-dose group exhibited a more modest rise at day 29 with a mean fold rise of 2.64 (ns, *p* = 0.081) compared to placebo (Fig. 5e, f, Supplementary Table 2). There were no significant differences in baseline saliva GII.4-specific IgA concentrations across groups (day 1 geometric mean [ng of specific/µg of total IgA]; placebo, 0.067; medium, 0.066; high, *p* ≥ 0.059). The highest GII.4-specific salivary IgA levels were observed at day 29 with mean fold rises of 1.78 (*p* = 0.021) and 1.84 (*p* = 0.0014) in the medium and high-dose groups compared to 1.03 in the placebo group (Fig. 5c, d, Supplementary Table 2). There were no significant differences in baseline nasal GII.4-specific IgA concentrations across groups (day 1 geometric mean [ng of specific/µg of total IgA]; placebo, 0.119; medium, 0.125; high, 0.093, p ≥ 0.14). The highest levels of GII.4-specific nasal IgA were observed at day 29 with mean fold rises of 5.96 (ns) and 6.31 (ns) in medium and high-dose groups compared to placebo with a mean fold rise of 0.95 (Fig. 5g, h, Supplementary Table 2). Together, these results demonstrate the presence of norovirus IgA in saliva and NLF in response to oral GI.1 and GII.4 vaccination.

## Discussion

Norovirus is a leading cause of AGE worldwide and poses a significant threat to vulnerable populations, particularly children in under-resourced regions with limited access to supportive care. In infants, norovirus infection can lead to severe dehydration requiring hospitalization and may result in death. Despite the significant need for a vaccine against norovirus, no licensed vaccine is currently available. Infants receive some protection from maternal antibodies transferred in utero, but these can wane within the first 5 months of life, leaving this population vulnerable to infection^23^. Current evidence suggests that consuming breast milk with baseline levels of norovirus-specific antibodies lessens disease severity, yet provides incomplete protection from infection and disease^13^. Previous work demonstrated that norovirus-specific fecal IgA generated after vaccination with VXA-G1.1-NN correlated with protection from infection and disease^12^. By vaccinating breastfeeding women, levels of norovirus-specific breast milk IgA could be boosted and passively transferred to the gastrointestinal tract of infants, providing or extending protection.

In this study, we report the safety and immunogenicity of a bivalent recombinant rAd5-vectored vaccine encoding the VP1 genes of norovirus GI.1 and GII.4 (VXA-G1.1-NN/VXA-G2.4-NS) administered orally to healthy breastfeeding women. The vaccine was well tolerated with only mild or moderate solicited AEs reported (35/76 participants [46.1% overall], 6/16 in placebo [37.5%] vs 29/60 in treatment groups [48.3%]). There was little difference in reported AEs between vaccine recipients and placebo recipients, with the most common AEs being headache and nausea. No other significant TEAEs of concern occurred over the subsequent 11 months. This study echoes the safety profile previously described for adults aged 18-80 years, where only mild to moderate reported AEs were observed^19,20,24^. Collectively, these results demonstrate that the safety profile of orally delivered norovirus VXA-G1.1-NN/VXA-G2.4-NS vaccines in adults aged 18-80 years extends to breastfeeding women.

The primary immunogenicity outcome, detecting vaccine-specific IgA in serum and breast milk, was achieved. Both GI.1 and GII.4-specific breast milk IgA reached a peak on day 29 and remained above background for up to six months for both treatment groups in the case of GI.1 and the high dose group in the case of GII.4. Meanwhile, GI.1 and GII.4-specific serum IgA levels peaked at day 8 post-vaccination and gradually decreased during the monitoring period.

Importantly, increases in norovirus-specific IgA compared to day 1 were detected in the stool of infants breastfed by the vaccinated participants but not placebo-treated participants. These antibodies were believed to originate from breast milk, as the norovirus-IgA content in breast milk and infant stool were significantly correlated. Interestingly, the placebo group displayed a significant correlation between breast milk and infant stool GII.4-specific IgA content, but not GI.1-specific IgA content. GII.4 was the most prevalent genotype from 2017-2019 in South Africa, whereas GI.1 was not observed^6^. Therefore, it is likely that baseline breastmilk GII.4-specific IgA was present and transferred to infants via breastfeeding regardless of vaccination status. Lastly, both breast milk and infant stool exhibited similar increases in norovirus-specific IgA1:IgA2 ratios at the peak response timepoint. These results suggest that norovirus-specific breast milk IgA can be induced by oral vaccination and can be passively transferred to infants, potentially extending increased protection to infants.

One approach to protecting infants from infectious diseases is through injected or IV-administered prophylactic monoclonal antibody therapy, the success of which is well-documented for respiratory syncytial virus^25^. However, beyond cost and cold-chain limitations, it is unlikely that this approach would be efficacious in preventing norovirus disease, as the sustained presence of intestinal IgA would likely be necessary to block virus infection and avert the rapid onset of symptoms. Alternatively, it is well established that breastfeeding can lessen diarrheal diseases and mortality, particularly in under-resourced areas^13,26^. Beyond providing a nutritional source, breast milk is a rich source of antibody-based immunity to gut pathogens, as antibody-secreting cells can migrate from the gut-associated lymphoid tissue (GALT) to the mammary glands^14,27^. A thermostable vaccine, such as the one described here, which can induce vaccine-specific breast milk antibodies for at least six months post vaccination, may provide an effective and easily deployable method of extending infant protection from AGE.

Vaccination of postpartum women has garnered support as a potential method of decreasing infant disease and mortality. Studies have identified vaccine-induced antibodies in breast milk against influenza^28,29^, pertussis^30^, and SARS-CoV-2^31^, among others. These studies have largely utilized vaccines given parenterally. While most vaccines are administered via injection, mucosal vaccination is expected to elicit a stronger mucosal immune response^32^. Peculiarly, in a direct comparison of influenza vaccines, the injected inactivated influenza vaccine (IIV) generated higher levels of breast milk antibodies than the intranasally-administered live-attenuated influenza vaccine (LAIV)^28,29^. In this case, it is unclear why a mucosally-delivered vaccine elicited lower breast milk antibody responses than its injected counterpart, although it is hypothesized that pre-existing nasal immunity in adults may limit LAIV replication and subsequent immune responses. Previous research on the rAd5 vector used in this study showed that immunogenicity was not limited by pre-existing anti-Ad5 immunity when administered orally^22^.

We previously demonstrated that this recombinant oral norovirus vaccine generated antibody responses at distal mucosal sites in older adults^12,19^ and antigen-specific serum IgG, serum IgA, and fecal IgA in young to middle-aged adults^20^. Here, we demonstrate that the generation of norovirus immunity can be extended to breastfeeding women, whereby a single vaccination resulted in norovirus-specific IgA in breast milk, serum, saliva, and NLF. The generation of mucosal antibody responses by the bivalent VXA-G1.1-NN/VXA-G2.4-NS vaccine is particularly valuable given that mucosal barriers are the first line of defense against norovirus. Beyond the passive transfer of norovirus-specific antibodies to breastfed infants, vaccination of breastfeeding women enhances the mother’s immunity, and possibly affords clinical protection, thereby reducing transmission risk between mother, child, and the larger community.

In addition to the practicality of delivering norovirus-specific antibodies to infants via breast milk, oral vaccination may stimulate pre-existing memory responses in vaccinated adults and generate more broadly cross-reactive antibodies, a response that could not be achieved by vaccination of naïve populations, like infants. Indeed, Park and colleagues previously identified a pan-GII-specific antibody from a participant vaccinated with this VXA-G2.4-NS^24^. Targeting the intestine through oral vaccination of adults previously exposed to norovirus may promote cross-genotype-specific responses by recalling memory B cells from previous exposures and boosting antibody repertoires that recognize conserved regions of norovirus capsids^24^. This finding suggests that cross-reactive memory responses may be generated by mucosal vaccination of participants with a history of exposure or infection, and these memory responses can be passively transferred to infants.

Curiously, the serum results described here differ from those reported in a previous clinical trial of a GI.1-only vaccine in adults aged 50-80 years, where serum antibody levels remained above background for up to seven months post-vaccination^19^. In this current study of breastfeeding women vaccinated with VXA-G1.1-NN/VXA-G2.4-NS, antibody levels remained elevated in breast milk after six months, but not in serum, saliva, or NLF. It is possible that while breastfeeding, IgA-producing antibody-secreting cells preferentially migrate to the mammary glands over other sites in the body^33^.

This study had a few important limitations. First, while we hypothesize that breast milk antibodies from oral vaccination could limit norovirus gastroenteritis in infants, we were unable to monitor for norovirus infection in participants or their infants. Future studies may enroll a larger population of mother-infant pairs and track the incidence of norovirus gastroenteritis. Likewise, increases in antibody responses due to environmental exposure, rather than vaccination, could not be discerned. However, observed increases in antibody responses in the treatment, but not the placebo groups, combined with temporal increases only after vaccination, support the immunogenicity of this vaccine. Lastly, participant immune responses were monitored six months post vaccination, although the WHO recommends breastfeeding through two years of age. Future studies may determine the long-term antibody levels of vaccinated participants and explore if a second dose extends or enhances immunity.

In this proof-of-concept study, we show that oral vaccination with the replication-incompetent rAd5-vectored vaccine was safe and well tolerated in post-partum breastfeeding women and induced serum, nasal, and salivary antibody responses. Importantly, we observed durable and functional antibody responses following oral vaccination in breast milk, and that breast milk antibodies were passively transferred to infants during breastfeeding. As infants are an especially vulnerable population and children are substantial carriers of norovirus, vaccination of breastfeeding women with an intestinal vaccine has the potential to impact both infant and community health. Some other oral enteric vaccines, like the live attenuated rotavirus vaccine, have shown reduced immunogenicity and efficacy in low- and middle-income countries (LMICs). It is thought that maternal rotavirus antibodies transferred in utero or via breast milk to the infant may interfere with vaccination and/or that gut microbiota dysbiosis may prevent oral vaccine uptake^34^. As opposed to live-attenuated vaccines, prior immune responses against the target antigen are unlikely to interfere with this vaccine’s uptake as the antigen of interest is conveyed by an rAd5 vector and doesn’t require on-going viral replication. Interestingly, the serum fold rise antibody responses trended slightly lower than that observed in US-based clinical trials^19^, which could be a function of diverse microbiota. Future studies may investigate the role of the microbiota in vaccine immunogenicity. Vaxart’s second-generation vaccine generates higher VP1 content in intestinal epithelial cells, which may improve immune recognition in the challenging intestinal environment^35^. This second-generation vaccine has already demonstrated improved immunogenicity in a phase 1 clinical trial (NCT06944717)^36^. Future studies will examine if this vaccination approach can limit infection, reduce disease severity, and prevent transmission to make a measurable impact on pediatric disease and mortality worldwide.

## Methods

### Study design, participants, and randomization

This study was a dose-ranging, phase 1, double-blind, placebo-controlled trial conducted across five sites in South Africa, examining the safety and immunogenicity of VXA-G1.1-NN/VXA-G2.4-NS administered as tablets to breastfeeding women. The trial was conducted by FARMVOS and sponsored by Vaxart, Inc., and the Bill and Melinda Gates Foundation. The trial protocol was approved by the South African Health Products Regulatory Authority (SAHPRA) and can be found under the South African National Clinical Trials Register (SANCTR, https://sanctr.samrc.ac.za/): DOH-27-072023-7893, registered on July 3^rd^ 2023 and additionally can be found under NCT07254728 on www.clinicaltrails.gov. Eligible participants were healthy, breastfeeding individuals assigned female at birth, aged 18 years or older, and with generally healthy infants aged one to 11 months with a gestational age of at least 28 weeks and without significant medical history or condition that could complicate breastfeeding. The paired infants were not considered enrolled participants in this study and did not receive the study vaccines. Inclusion criteria included an intention to continue breastfeeding for at least one month with the goal to continue for at least six months post vaccination. Exclusion criteria included active infections (human immunodeficiency virus, hepatitis B virus, or hepatitis C virus), conditions or medications that might impact the immune response to the vaccine, or a gastrointestinal condition or medication that could affect the absorption and immune response to an orally administered vaccine. All participants provided written informed consent. Eligible participants were randomized at approximately 2:2:1 into treatment groups of 5 × 10^10^ IU/genotype or 1 × 10^11^ IU/genotype or administered placebo tablets (Fig. 1a). The sample sizes were determined based on clinical judgment; no formal sample size or power calculation was conducted. Randomization was managed via the Medidata System and an unblinded pharmacist placed the treatment in a secondary container for delivery to the blinded clinical team for administration to participants. No concomitant care was administered by the sites during the study period. Participants and investigators were blinded to treatment assignment. Participants were observed for 30 min after study drug administration for any acute reactions. Local and systemic reactogenicity were solicited from participants for seven days after administration, and unsolicited treatment-emergent adverse events (TEAEs) were recorded for 29 days after administration. Participants were followed for one year following study tablet administration, and any serious adverse events (SAEs), adverse events of special interest (AESIs), and new onset of chronic illnesses (NOCIs) were recorded (Fig. 1b).

### Vaccine production

VXA-G1.1-NN/VXA-G2.4-NS is an E1/E3-deleted replication-incompetent recombinant adenovirus type 5 that expresses the VP1 gene of norovirus genotypes GI.1 and GII.4 along with a double-stranded ribonucleic acid hairpin adjuvant. It was produced in Expi293F cells in accordance with Good Manufacturing Practice (GMP) at Vaxart, Inc. in South San Francisco, CA, as previously described^20^. The final drug product was formulated into enterically coated tablets for single administration.

### Study groups and statistical analysis

All participants who completed dosing were analyzed for safety and reactogenicity. Participants with at least one valid pre-vaccination and one post-vaccination clinical sample dose were analyzed for immunogenicity endpoints, except for infant stool, where samples collected within one week of vaccination were included as a pre-vaccination clinical sample. Immunogenicity results were analyzed using mixed-effect analysis with multiple comparisons, and Uncorrected Fisher’s Least Significant Difference (LSD) tests unless otherwise noted. All data were analyzed using GraphPad Prism Version 10.4.1. Pearson or Spearman correlations were run as indicated in the figures. No interim analyses were planned or performed.

### Breast milk processing and MSD assays

Breast milk skim was isolated by centrifugation at 3000 x g at 4 °C. The middle skim layer was transferred to a new tube and centrifuged again at 3000 x *g* at 4 °C. The middle skim layer was stored at −80°C. Antigen-specific breast milk IgA was measured by coupling biotinylated GI.1 and GII.4 VLPs (AscentGene, Inc.) at a concentration of 66 nM to Meso Scale Discovery (MSD) linkers, which were added to MSD U-PLEX 2-Assay, 96-Well SECTOR Plates (MSD, K15227N) and incubated for one hour at room temperature (RT). The plates were washed 3x with PBST (1X PBS with 0.001% Tween-20) and blocked with 3% Amersham ECL Blocking Agent in PBST (Cytiva, RPN2125) for one hour at RT, followed by another wash. Skim samples were diluted at 1:100 and 1:500 in 1% Blocking Agent/PBST and incubated on the plates for two hours at RT with shaking. Wells were incubated with 50 µL of Sulfo-Tag Anti-Hu/NHP IgA Antibody (MSD, D20JJ-6) diluted to 1 μg/mL in 1% Blocking Agent/PBST for one hour at RT, then washed and incubated with 50 μL/well of MSD GOLD Read Buffer B (MSD, R60AM-2). The plates were read on a Meso QuickPlex SQ 120MM instrument, and antigen-specific IgA signals are reported in RLU. Total IgA content was quantified by adding 50 μL/well of anti-human IgA monoclonal antibody at 1 μg/mL (Mabtech, 3860-6000) to MSD GOLD 96-well small spot streptavidin plates (MSD, L45SA-1) and incubating for one hour/RT. The plates were washed with PBST. Wells were incubated with 50 µL of breast milk skim diluted at 1:5000 and 1:10000 in 1% Blocking Agent/PBST or a standard curve of purified human secretory IgA (Bio-Rad, PHP133) ranging from 0.7813 - 1600 ng/mL for two hours/RT. Plates were washed, and Sulfo-Tag Anti-Hu/NHP IgA Antibody (MSD, D20JJ-6) was added as above. Plates were washed, and 50 μL/well of Read Buffer A (MSD, R92TG-4) was added to the plates and read as above. Antigen-specific breast milk IgA was normalized to the total amount of IgA in the corresponding sample for final values of relative light units (RLU)/microgram of total IgA. Fold rise was calculated by dividing the normalized antigen-specific breast milk IgA values at each time point by the day 1 values.

### Breast milk antibody purification and quantification

Approximately 500 μL of processed skim from selected medium and high dose participants was diluted 1:5 in 1X PBS and incubated with CaptureSelect IgA-CH1 (Thermo Scientific, 194311005) for two hours with rotation. Antibody:bead complexes were collected by centrifugation and washed 2X with 1X PBS. Antibody was eluted in 450 μL of elution buffer [0.1 M glycine (pH 3)], and eluant was adjusted to a neutral pH with 1 M Tris-HCl pH 7. Total antibody concentration was determined using small spot streptavidin plates as described above.

### Norovirus blocking antibody assay (NBAA)

The NBAA has been previously described^15^. Briefly, NeutrAvidin-coated plates (Thermo Fisher Scientific) were washed with Wash Buffer (0.1 M sodium phosphate, pH 6.4), and either biotinylated Le^b^ or H Type 3 HBGAs (2.5 μg/mL) were immobilized on the plates. Biotinylated GI.1 or GII.4 VLPs (AscentGene Inc.) were incubated with serial dilutions of purified sample at 37 °C/1 h. Mixtures were then transferred to HGBA-coated plates and incubated at 4 °C/2 h. Plates were washed with Wash Buffer and incubated with rabbit anti-GI.1 or anti-GII.4 VLP polyclonal antisera (Thermo Fisher Scientific) at 4 °C/1 h. Bound VLPs were detected with Goat anti-rabbit IgG-HRP (Bethyl Laboratories, A120-101P) after incubation at 4 °C/1 h. Plates were washed, and colorimetric development was initiated with 100 μL/well of TMB substrate (Rockland Inc., TMBE-1000). Absorbance was measured at 450 nm using the Agilent BioTek Cytation 7 plate reader or similar. NBAA titer was defined as the highest dilution factor yielding an OD value less than 25% (breast milk antibodies) or 50% (serum) of the VLP-only controls OD values. Results below the lowest dilution (breast milk, 1:4; serum, 1:25) are reported as half the dilution. Serum NBAAs were performed at PPD, Inc. under qualified conditions, and breast milk NBAAs were performed at Vaxart. For purified breast milk antibodies, NBAA titer/µg of total antibody was calculated by dividing the NBAA titer value by the microgram amount of total antibody present in 50 μL of sample.

### Serum IgA and IgG MSD assays

The assay was performed in-house based on an assay format previously qualified by PPD, Inc.^15^ GI.1 and GII.4 VLP were linked to MSD plates as described above. Serum was diluted 1:1600 in 1% ECL Blocking Agent (Cytiva, Marlborough, MA, USA) in 1X PBST and incubated at RT for one hour, shaking at 700 rpm. A qualified standard pre-screened for high levels of antibodies to norovirus was serially diluted 4-fold and included in all assays starting at a 1:25 dilution. Plates were washed with 1X PBST and incubated with 1 µg/mL solutions of MSD SULFO-TAG anti-IgG or anti-IgA for 1 h, shaking at 700 rpm. Plates were washed with 1X PBST and developed using MSD Gold Read Buffer B. Data was acquired using the MSD Sector Imager 120 instrument and expressed as MSD arbitrary units (AU)/mL.

### IgA detection in nasal lining fluid and saliva by MSD

Nasosorption FX-i devices (Mucosal Diagnostics) were used to collect nasal lining fluid (NLF) from the left and right nasal cavity according to manufacturer’s instructions. The right and left absorbent matrixes of the Nasosorption FX-i devices were immediately stored at -80°C. NLF was collected by thawing the matrixes at RT and the absorbent tips were placed into a designated well on a Chromtech 96-well plate. 700 μL of elution buffer (PBST 1% BSA) was added to each well, vortexed, and the plate was centrifuged at 1000 x *g* for 15 min. The resulting nasal eluant was stored at −80 °C. Antigen-specific nasal IgA was measured as described above using dilutions of 1:5 and 1:10 and run along an antigen-specific standard curve (custom) to determine ng quantities of antigen-specific antibodies. Total IgA content was run as described above using dilutions of 1:1000 and 1:5000. Antigen-specific nasal IgA was normalized to the total amount of IgA in the corresponding sample to get final values of ng antigen-specific IgA/µg total IgA. Fold rise was calculated by dividing the normalized antigen-specific nasal IgA values at each time point by the day 1 values. A similar process was used to detect salivary IgA with the following modifications: A salivette (Sarstedt, 51.1534) was used to collect saliva on given days according to manufacturer’s instructions. Saliva was eluted by centrifugation at 4˚ /1000 x *g*/2 min. Specific and total IgA were quantified as described above.

### Stool supernatant processing for ELISAs

Whole infant stool (200 mg) was transferred to a 2 mL microcentrifuge tube containing 500 mg of 2.3 mm zirconia silica beads and 1 mL extraction buffer (soybean trypsin inhibitor (STI), ethylenediaminetetraacetic acid (EDTA) solution, 1X Phosphate Buffered Saline, pH 7.4 + 0.05% Tween-20 (PBST), phenylmethanesulfonylfluoride (PMSF) solution). Stool specimens were homogenized for 1 min, followed by centrifugation at 14,000 × *g*/30 min at 4 °C. The resulting supernatants were then collected, stabilization-preservative buffer (10% bovine serum albumin [BSA]) in PBS and 1% sodium azide in sterile water was added at a 1:50 ratio, and supernatants were stored at −80 °C until they were assayed.

### IgA detection in stool by ELISA

Stool ELISAs were performed at the University of Maryland School of Medicine. Medium-binding microtiter plates (Greiner Bio-One) were coated with either 1 μg/mL purified goat anti-human IgA (α-chain specific) (Jackson ImmunoResearch) for total IgA or 0.5 μg/mL of either GI.1 or GII.4 VLPs (AscentGene) for norovirus-specific IgA, all diluted in 1X PBS, pH 7.4. All plates were incubated overnight at 4°C. After coating incubation, plates were washed three times with PBST. Blocking buffer (PBST + 5% non-fat dry milk [NFDM] [Nestlé]), was added to all wells, and plates were incubated for 1 h at 37°C. Samples, standards, and assay controls were diluted in blocking buffer. Stool supernatant samples were diluted at 1:2 and 1:10,000 for specific and total IgA, respectively. Total IgA was quantified with human IgA purified from plasma (Calbiochem) that was serially diluted twofold from 15.4 ng/mL to generate a seven-point standard. An assay-specific norovirus IgA-positive in-house standard was serially diluted and included independently for specific IgA assays. Clinical samples, standards, controls, and diluent-only blanks were added to plates in duplicate, and plates were incubated for 1 h at 37 °C. Plates were then washed as described above, and horseradish peroxidase (HRP)-labeled α-specific goat human IgA (Jackson ImmunoResearch) was diluted 1:5,000 in PBST + 5% NFDM and added to all wells for each assay. Plates were incubated for another 1 h at 37 °C. TMB Microwell Peroxidase Substrate (SeraCare) was added, and plates were incubated for 15 min in the dark with agitation at ambient temperature. The colorimetric reaction was stopped with the addition of 1 M phosphoric acid to all wells. Absorbance, measured as optical density at 450 nm (OD_450_), was read using a Multiskan FC™ Microplate Reader (Thermo Scientific). Blank signal was subtracted from values, and OD_450_ of the standards was analysed using four-parameter logistic (4PL) regression in GraphPad Prism. Total and specific IgA concentrations (µg/mL) in clinical samples were interpolated and multiplied by the on-plate sample dilution. Results were reported as the ratio of norovirus-specific IgA to total IgA. Samples for which IgA concentrations were poor were excluded from analysis (three individual samples).

## Supplementary information

Supplementary Figures and Tables

## Data Availability

This trial was registered with the South African National Clinical Trials Register (trial reference DOH-27-072023-7893). All relevant data is contained within the manuscript main text and supplemental materials. The raw immunological data of deidentified participants is available upon request to the corresponding author.
