# Supplementary material for: Transfer of breast milk IgA to infants after oral bivalent norovirus vaccination of post-partum women
Source: NPJ Vaccines. 2026 Jan 14;11:44. doi: 10.1038/s41541-025-01361-0 (PMC12887003; doi:10.1038/s41541-025-01361-0)
Supplement: Supplementary file 1 — Supplementary Figures and Tables [file 41541_2025_1361_MOESM1_ESM.pdf]

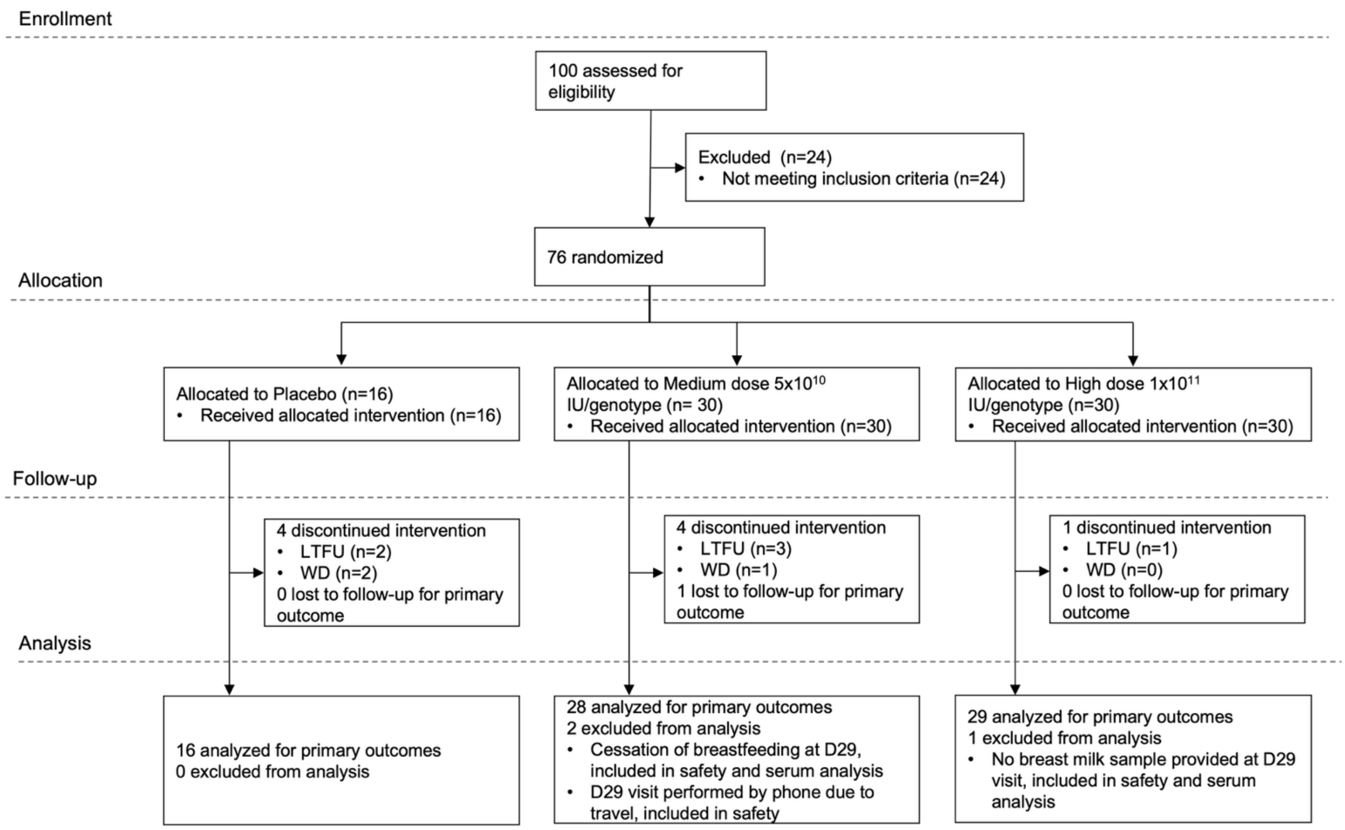

Supplementary Figure 1: Trial profile, enrollment, and analysis. Abbreviations: IU, infectious units, LTFU=Lost to follow up, WD=withdrew consent.

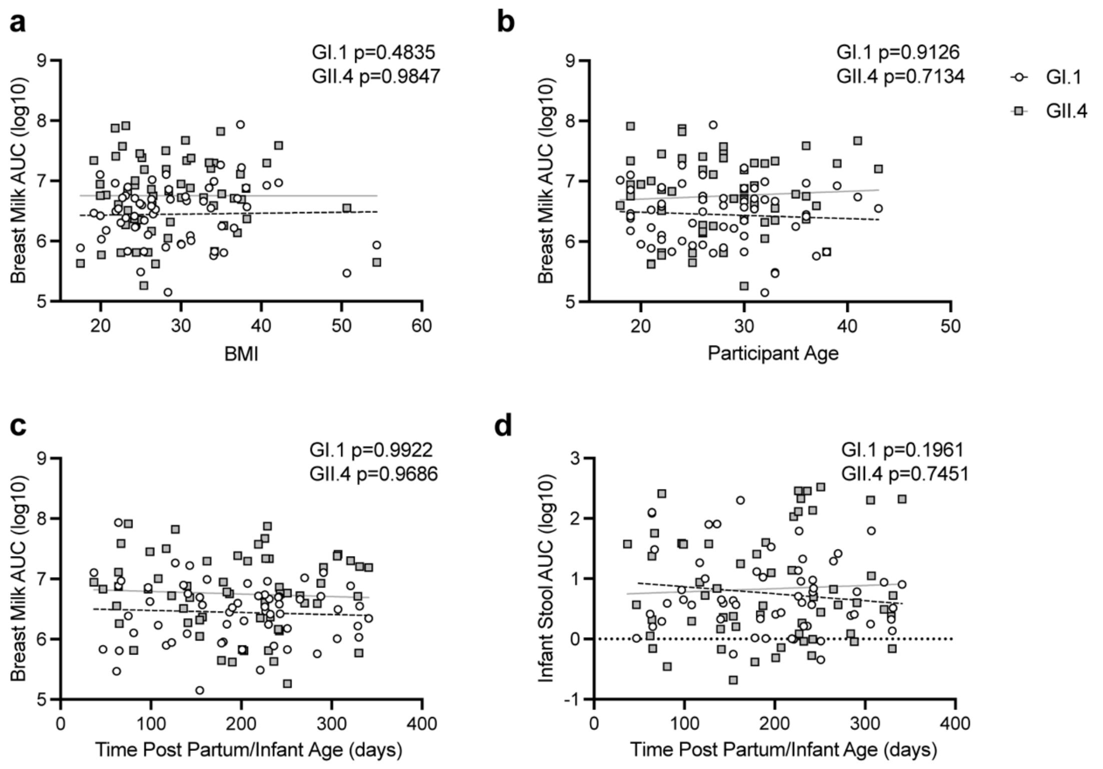

Supplementary Figure 2: Demographic characteristics did not influence breast milk immunogenicity. Breast milk AUC from days 1-60 of medium and high dose group were calculated and plotted against (A) BMI, (B) Age, or (C) Time post-partum. (D) Infant stool AUC from days 1-60 of medium and high dose group were calculated and plotted against infant age. Simple linear regression and Pearson correlation shown, n=60 for breast milk and n=59 for infant stool. Abbreviations: AUC=area under the curve, BMI=body mass index.

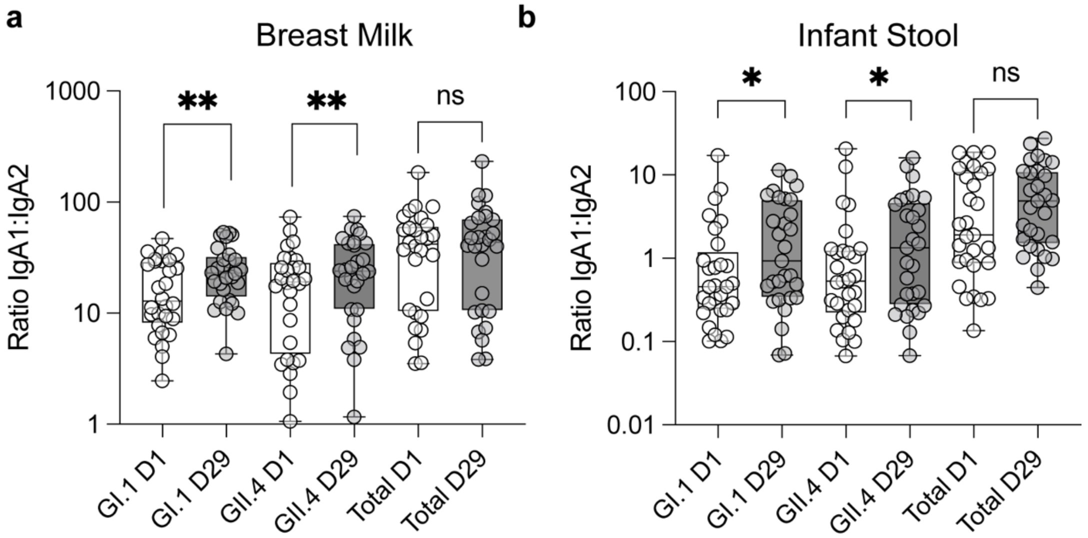

Supplementary Figure 3: Dynamics of antigen-specific IgA1 to IgA2 ratios over time was mirrored between breast milk and infant stool. (A-B) RLUs of IgA1 and IgA2 were determined for breast milk and infant stool in samples from the high dose group and plotted as ratios. Median and interquartile range shown with a Wilcoxon paired t-test, n=30. Abbreviations: RLU=relative light units.

Supplementary Table 1. **Unsolicited treatment emergent adverse events (TEAE) from day 1 (dose) to 29.**

	**Placebo cohort (N = 16)**	**Medium dose cohort (N = 30)**	**High dose cohort (N = 30)**	**Medium + high dose cohorts (N = 60)**	**Overall (N = 76)**
Number of Subjects Reporting at least one:					
Unsolicited TEAE, participants affected, n (%)	2 (12.5%)	3 (10.0%)	3 (10.0%)	6 (10.0%)	8 (11.8%)
Unsolicited TEAE, number of events, n	2	3	5	8	10
Related TEAE[1], participants affected, n (%)	2 (12.5%)	1 (3.3%)	1 (3.3%)	2 (3.3%)	4 (5.3%)
Related TEAE[1], number of events, n	2	1	1	2	4
Severe TEAE (Grade ≥ 3)[2], n (%)	0	0	0	0	0
Serious TEAE, n (%)	0	0	0	0	0
AESI[3], n (%)	0	0	0	0	0
NOCI[4], n (%)	0	0	0	0	0
Unsolicited TEAEs lead to study drug discontinuation, n (%)	0	0	0	0	0
Unsolicited TEAEs result in death, n (%)	0	0	0	0	0

[1]Related TEAE is defined as a relationship to study drug as ‘possibly related’, ‘probably related’, or ‘definitely related’.
[2]Grade 1: Mild, asymptomatic or mild symptoms; Grade 2: Moderate, minimal, local or noninvasive intervention indicated; Grade 3: Severe, medically significant but not immediately life-threatening; Grade 4: Life-threatening consequences, urgent intervention indicated; Grade 5: Death related to AE. Abbreviations: AESI, adverse events of special interest; NOCI, new onset of chronic illness; TEAE, treatment-emergent adverse event.

Supplementary Table 2: Mean fold rise and SEM of immunogenicity endpoints.

	GI.1								
Breast Milk IgA	Placebo			Medium			High		
	Mean	SEM	N	Mean	SEM	N	Mean	SEM	N
D1	1.00	0.00	16	1.00	0.00	30	1.00	0.00	30
D8	1.13	0.10	16	3.43	1.96	30	2.16	0.34	30
D29	1.40	0.35	16	5.57	2.46	28	4.04	1.00	29
D60	0.91	0.15	16	3.05	0.96	29	3.23	0.84	29
D180	1.35	0.48	16	2.28	0.66	27	2.08	0.57	27
Infant Stool IgA[1]	Placebo			Medium			High		
	Geo Mean	Upp. Limit	Low. Limit	Geo Mean	Upp. Limit	Low. Limit	Geo Mean	Upp. Limit	Low. Limit
D1	1.00	1.00	1.00	1.00	1.00	1.00	1.00	1.00	1.00
D29	1.19	1.91	0.74	3.29	6.87	1.57	2.01	3.66	1.11
D60	0.72	1.16	0.44	2.47	4.64	1.32	1.68	2.70	1.05
Serum IgA	Placebo			Medium			High		
	Mean	SEM	N	Mean	SEM	N	Mean	SEM	N
D1	1.00	0.00	16	1.00	0.00	30	1.00	0.00	30
D8	1.44	0.50	16	7.41	2.29	30	5.63	1.47	30
D29	1.85	0.63	16	3.42	0.86	29	2.68	0.44	30
D180	1.33	0.39	16	2.13	0.75	28	1.30	0.20	28
Serum IgG	Placebo			Medium			High		
	Mean	SEM	N	Mean	SEM	N	Mean	SEM	N
D1	1.00	0.00	16	1.00	0.00	30	1.00	0.00	30
D8	1.50	0.52	16	2.32	0.69	30	2.57	0.50	30
D29	1.73	0.61	16	3.38	0.98	29	5.06	1.26	30
D180	2.99	1.18	16	1.98	0.39	28	2.69	0.59	28
Saliva IgA	Placebo			Medium			High		
	Mean	SEM	N	Mean	SEM	N	Mean	SEM	N
D1	1.00	0.00	16	1.00	0.00	29	1.00	0.00	29
D8	1.17	0.10	16	1.52	0.19	29	1.44	0.19	29
D29	1.14	0.13	16	2.05	0.38	28	1.33	0.13	28
D60	1.60	0.23	16	2.72	0.47	29	1.71	0.34	28
D180	1.53	0.25	16	2.19	0.33	27	2.19	0.69	27
NLF IgA	Placebo			Medium			High		
	Mean	SEM	N	Mean	SEM	N	Mean	SEM	N
D1	1.00	0.00	16	1.00	0.00	30	1.00	0.00	30
D8	0.85	0.14	16	3.56	1.32	30	1.56	0.23	30
D29	1.05	0.16	16	4.86	1.91	29	2.64	0.86	29
D60	0.75	0.16	16	1.36	0.26	30	2.31	0.87	29
D180	0.90	0.26	16	1.52	0.31	28	2.15	1.08	28

	GII.4								
Breast Milk IgA	Placebo			Medium			High		
	Mean	SEM	N	Mean	SEM	N	Mean	SEM	N
D1	1.00	0.00	16	1.00	0.00	30	1.00	0.00	30
D8	1.18	0.10	16	1.24	0.14	30	2.30	0.48	30
D29	1.08	0.10	16	2.89	1.15	28	5.99	1.56	29
D60	0.83	0.09	16	2.73	0.95	29	4.05	1.18	29
D180	0.87	0.12	16	1.13	0.30	27	2.98	0.92	27
Infant Stool IgA[1]	Placebo			Medium			High		
	Geo Mean	Upp. Limit	Low. Limit	Geo Mean	Upp. Limit	Low. Limit	Geo Mean	Upp. Limit	Low. Limit
D1	1.00	1.00	1.00	1.00	1.00	1.00	1.00	1.00	1.00
D29	0.71	1.02	0.49	1.22	2.30	0.64	4.10	8.81	1.91
D60	0.65	1.07	0.39	0.99	1.80	0.54	2.05	4.26	0.99
Serum IgA	Placebo			Medium			High		
	Mean	SEM	N	Mean	SEM	N	Mean	SEM	N
D1	1.00	0.00	16.00	1.00	0.00	30.00	1.00	0.00	30
D8	1.53	0.55	16.00	3.32	0.86	30.00	4.66	0.87	30
D29	1.57	0.54	16.00	1.96	0.34	29.00	3.59	1.06	30
D180	2.09	0.66	16.00	1.22	0.20	28.00	1.78	0.41	28
Serum IgG	Placebo			Medium			High		
	Mean	SEM	N	Mean	SEM	N	Mean	SEM	N
D1	1.00	0.00	16.00	1.00	0.00	30.00	1.00	0.00	30
D8	1.55	0.58	16.00	1.11	0.05	30.00	2.10	0.44	30
D29	1.51	0.61	16.00	1.14	0.11	29.00	3.35	1.12	30
D180	2.44	1.07	16.00	1.07	0.14	28.00	1.90	0.44	28
Saliva IgA	Placebo			Medium			High		
	Mean	SEM	N	Mean	SEM	N	Mean	SEM	N
D1	1.00	0.00	16.00	1.00	0.00	29.00	1.00	0.00	29
D8	1.16	0.12	16.00	1.20	0.14	29.00	1.56	0.14	29
D29	1.03	0.09	16.00	1.78	0.29	28.00	1.84	0.21	28
D60	1.19	0.11	16.00	1.81	0.25	29.00	1.48	0.18	28
D180	1.31	0.18	16.00	1.33	0.16	27.00	1.62	0.25	27
NLF IgA	Placebo			Medium			High		
	Mean	SEM	N	Mean	SEM	N	Mean	SEM	N
D1	1.00	0.00	16.00	1.00	0.00	30.00	1.00	0.00	30
D8	0.88	0.12	16.00	2.83	1.47	30.00	1.77	0.29	30
D29	0.95	0.13	16.00	5.96	4.14	29.00	6.31	3.35	29
D60	0.54	0.12	16.00	0.97	0.20	30.00	2.50	0.97	29
D180	0.53	0.07	16.00	1.06	0.23	28.00	2.10	0.78	28

[1]Geometric mean and 95% confidence interval was used for infant stool due increased variability in the samples.
Abbreviations: NLF=nasal lining fluid.
